# Expert Recommendations on the Evaluation of Sunscreen Efficacy and the Beneficial Role of Non-filtering Ingredients

**DOI:** 10.3389/fmed.2022.790207

**Published:** 2022-03-31

**Authors:** Salvador González, José Aguilera, Brian Berman, Piergiacomo Calzavara-Pinton, Yolanda Gilaberte, Chee-Leok Goh, Henry W. Lim, Sergio Schalka, Fernando Stengel, Peter Wolf, Flora Xiang

**Affiliations:** ^1^Medicine and Medical Specialties Department, University of Alcalá de Henares, Madrid, Spain; ^2^Dermatological Photobiology Laboratory, Medical Research Center, School of Medicine, University of Málaga, Málaga, Spain; ^3^Department of Dermatology and Cutaneous Surgery, University of Miami-Florida, Miami, FL, United States; ^4^Department of Dermatology, University of Brescia, Brescia, Italy; ^5^Department of Dermatology, Hospital Universitario Miguel Servet, IIS Aragón, Zaragoza, Spain; ^6^National Skin Centre, Singapore, Singapore; ^7^Department of Dermatology, Henry Ford Health System, Detroit, MI, United States; ^8^Photoprotection Laboratory, Medicine Skin Research Center, São Paulo, Brazil; ^9^Buenos Aires Skin, Buenos Aires, Argentina; ^10^Department of Dermatology, Medical University of Graz, Graz, Austria; ^11^Department of Dermatology, Shanghai Medical College, Huashan Hospital, Fudan University, Shanghai, China

**Keywords:** sunscreen, photoprotection, UV, ISO, skin immunity, polypodium leucomotos

## Abstract

A variety of non-filtering agents have been introduced to enhance sunscreen photoprotection. Most of those agents have only weak erythema protective properties but may be valuable and beneficial in supporting protection against other effects of UV radiation, such as photoimmunosuppression, skin aging, and carcinogenesis, as well as photodermatoses. The question arises how to measure and evaluate this efficacy since standard SPF testing is not appropriate. In this perspective, we aim to provide a position statement regarding the actual value of SPF and UVA-PF to measure photoprotection. We argue whether new or additional parameters and scales can be used to better indicate the protection conferred by these products against the detrimental effects of natural/artificial, UV/visible light beyond sunburn, including DNA damage, photoimmunosuppression and pigmentation, and the potential benefits of the addition of other ingredients beyond traditional inorganic and organic filters to existing sunscreens. Also, we debate the overall usefulness of adding novel parameters that measure photoprotection to reach two tiers of users, that is, the general public and the medical community; and how this can be communicated to convey the presence of additional beneficial effects deriving from non-filtering agents, e.g., biological extracts. Finally, we provide a perspective on new challenges stemming from environmental factors, focusing on the role of the skin microbiome and the role of air pollutants and resulting needs for photoprotection.

## Sun Protection Factor and UVA Protection Factor: Are They Still the Benchmark for Photoprotection?

Population growth, increasing awareness of the consequences of sun exposure and increased lifespan, together with the increased incidence of sun-related skin tumors have resulted in photoprotection becoming indispensable (1). The choice of sunscreens and their proper use have become extremely important, forming the basis of a corner of the pharmaceutical market with enormous economic impact. However, this investment and expense does not correlate with a decrease in the incidence of skin cancer. This means that there are fundamental gaps in the manner photoprotection is measured, communicated and applied by scientists and companies to guide sunscreen usage by the final users, i.e., the general public.

The current standards are the SPF and UVA-PF parameters. Other normatives cover related issues, e.g., sunscreen water resistance (ISO 16217:2020: Cosmetics—Sun protection test methods—Water immersion procedure for determining water resistance).

The FDA states: “SPF is a measure of how much solar energy (UV radiation) is required to produce sunburn on protected skin (i.e., in the presence of sunscreen) relative to the amount of solar energy required to produce sunburn on unprotected skin. As the SPF value increases, sunburn protection increases.” The International Organization for Standardization (ISO) dictated a regulatory norm (ISO24444:2019), “Cosmetics—Sun protection test methods—*in vivo* determination of the sun protection factor (SPF)” that covers the testing methods, reference values and every parameter related to the ability of a given substance to act as a photoprotector. In practice, the effectiveness of photoprotection achieved by topical formulations is much more difficult to evaluate than expected. Several parameters need to be taken into account, including seasonal, meteorological and geographical considerations; skin phototype; amount of sunscreen applied; and frequency of re-application. None of these parameters is absolutely precise in every condition, and for every individual. As a result of this variability, many end users are much less protected than they believe.

A key value for *in vivo* determinations is the Minimal Erythema Dose (MED), which is defined as the threshold dose of solar radiation that produces sunburn. In fact, the SPF (Sun Protection Factor) that appears as a number (usually between 3 and 50+, or in some markets even more) on every sunscreen container is a ratio between the MED of skin treated with sunscreen divided by the MED of untreated skin. For example, if a sunscreen has a SPF value of 10, it means that it takes 10 times longer to induce erythema in treated skin compared to unprotected skin. It is thus obvious that the dose is a crucial factor to define the given MED of a photoprotector. The required dose of sunscreen applied for testing procedures is 2 mg/cm^2^.

Another important parameter is the UVA protection Factor (UVA-PF), which is obtained from *in vitro* measurements. Based on the determination of UVA (320–400 nm) transmittance in methacrylate or PMMA plates coated with 1.3 mg/cm^2^ sunscreen (ISO24443:2012, updated recently to ISO24443:2020), UVA-PF reflects *in vivo* protection conferred by the sunscreen against UVA-induced persistent pigment darkening (at 2–4 h after exposure). A sunscreen with an Ultraviolet A (UVA)-PF of 10 indicates that, e.g., in a subject with skin phototype IV it takes 10 times longer to develop PPD in sunscreen-protected skin compared to unprotected skin.

However, the manner in which some ISO-standardized measurements are made allows predicting the SPF and UVA-PF. The procedure consists of applying the sunscreen on artificial skin models and measuring the spectral transmittance at different wavelengths of the Ultraviolet (UV) spectrum. Although these systems are not included in ISO24444:2019, they provide measurements sufficiently comparable to those obtained from human volunteers, with some caveats described below. There is normalized precedent for this, as ISO 24443:2012 describes the *in vitro* determination of photoprotection against UVA using a hybrid method that relies on *in vitro* measurements but requires the SPF *in vivo* data for its determination. Although the FDA accepts the critical wavelength method, also *in vitro* and with an execution method similar to ISO 24443, the method has not yet been shown to be reproducible and interchangeable with SPF *in vivo*, especially in high SPF formulations.

Other alternative examples include the COLIPA method (Guidance drafted by The European Cosmetics Association). This method assesses UV transmittance of a thin layer of sunscreen on a PMMA roughened substrate after exposure to a controlled dose of UV radiation from a defined UV source (2). However, it has been shown that SPF fluctuates in a roughness-dependent manner (3). A similar method developed by the National Institute of Public Health (NIPH) measures attenuation of UVB intensity on a defined layer of a sunscreen product irradiated with an UVA/UVB source, a sheet of Mikelanta covering paper with 2 mg/cm^2^ of product and assessed by a radiometer (4). The VUOS method employs surgical tape affixed to a quartz layer with 1.2 mg/cm^2^ of product applied, and SPF calculation from transmittance measurements (5). Another method uses diffusing plates made of quartz fixed with surgical adhesive tape on human skin biopsies (6). This enables obtaining very precise spectra that define the photoprotective efficiency of a sunscreen at different UV wavelengths. The FDA (United States) uses the critical wavelength as a means to assess broad spectrum protection. Critical wavelength is the wavelength below which 90% of the area under the absorption curve resides. For products to be eligible for “broad spectrum” label, the critical wavelength must be ≥370 nm. Finally, other methods could be incorporated into future ISO covering the determination of photoprotective properties, for example, Hybrid Diffuse Reflectance Spectroscopy (HDRS) is a promising *in vivo* alternative due to the fact that it does not require erythema induction, measuring instead skin reflectance. HDRS displays excellent correlation with ISO24444:2019 (7).

The FDA recently proposed that UV filters approved in the US to be categorized into three categories, based on determination of GRASE (Generally Recognized as Safe and Effective). The list of FDA-approved GRASE products is very short, including only TiO_2_ and ZnO (8). Two filters are categorized as not GRASE, while additional safety data have been requested for the remaining filters (9) in order to be considered GRASE. Conversely, the EU considers sunscreens as cosmetics, thus additional substances are allowed.

However, at the consumer level these considerations are much less relevant. The most recognized parameter by the general public is the SPF. However, higher SPF leads to a false sense of protection because quantities applied are usually much lower than needed and therefore the “true” SPF is much lower. Considering that a normal-sized adult skin measures 2 m^2^, an adult whose height is 1.73 m would require 35 g of sunscreen per application (10), which a very small percentage of the population actually employs. Furthermore, the actual amount of effective sunscreen on the skin is reduced due to the routine habits that accompany the usual need for photoprotection, e.g., friction with clothing, sweat, water immersion, etc., which decreases the *de facto* amount of sunscreen that remains on the skin post-application. Therefore, the consumer acts according to this number, generally thinking that if the number is high enough, they can disregard additional safety issues, including avoiding midday exposure, seeking shade when outdoors, limiting exposure time, wearing additional protective measures (clothes, hats, etc.), and the need for frequent reapplication of the product.

In addition to this crucial issue, SPF does not account for additional damage caused to the skin beyond MED-related measurements. The most crucial issues are DNA damage, photoaging and immunosuppression. There is a large (and growing) body of evidence indicating that sub-MED doses of UV radiation, particularly by longer exposure to low energy (UVA) photons, accelerate photoaging and mediate immunosuppression and DNA damage. Some of these effects are related to DNA-induced damage, and some are due to deletion of specific cell subpopulations. Although parameters to measure these effects do exist (see below), they are not incorporated into the ISO standard SPF or UVA-PF parameters. Finally, neither the ISO rules nor the FDA monograph on the topic take into consideration the effects of visible (VL) and infra-red (IR) light, and a growing body of evidence indicates that these wavelengths also produce biological effects on different cellular skin populations (11–13). In this article, we posit that there is an emergent need for complementary methods that address photoprotective needs and measures beyond erythema, which is the focus of SPF measurements, particularly given the steady increase in diagnosed skin cancer cases over the past 10 years, which are not necessarily related to erythematous reactions. In addition, future determinations of photoprotective ability would ideally be performed *in vitro*, which would reduce variability and prevent human subjects from receiving high doses of light in the UV and other ranges. Other wavelengths are important given the increasing cases of photodermatosis and photosensibilization, which are important at visible and IR ranges. The major challenge will be to integrate the information conveyed by SPF measurements with these new methods. Ideally, the research community should strive to provide a comprehensive final index that is easily understandable by end-users. Such index would include ISO-compliant SPF measurements as well as additional information regarding the positive biological effects of other components of the sunscreen formulation.

Sunscreens may also contribute to the process of photo-adaptation, which consists to decreased erythema and inflammation in response to acclimation during repeated exposure (14). This is a common phenomenon that may reduce the danger of developing cancer, but may also affect specific responses to therapy (15, 16).

## UV-Induced DNA Damage

UV radiation causes DNA damage (17). The best characterized effects of UV photons on DNA include the formation of thymine and pyrimidine-pyrimidone dimers (18, 19) and 8-hydroxy-2′-deoxyguanosine (8-oxo-dG), which is an oxidized derivative of deoxyguanosine also involved in carcinogenesis (20). DNA damage is often measured as the appearance of the H2A histone family member X (H2AX) (21). Normally, the onset of these types of DNA damage would cause apoptosis, but the targeting of specific tumor suppressor such as p53 enables the survival of these cells (22, 23), which become tumor seeds. UV radiation also induces telomere shortening and degradation (24). These effects may cause transforming DNA mutations (25), leading to the emergence of photo-induced carcinogenesis.

Although high energy UV photons are the most important triggers of these alterations (26), lower energy UVA photons, or even visible light photons, may also induce these effects (27, 28). This is due, at least in part, to the oxidative stress (generation of ROS) induced by UV and visible photons (27, 29). In addition to damaging DNA, ROS also trigger diverse signaling pathways involved in cell proliferation, e.g., the MAPK pathway, JNK/p38, expression of AP1 and COX2 and activation of the NF-kB pathway (30–32). In addition, UV radiation affects the function of Nrf2, which is a controlling hub for antioxidant response by determining the expression of natural antioxidant enzymes, including Glu-6-phosphate dehydrogenase, thioredoxin reductase, glutathione S-transferase, and peroxidase (33). Finally, mitochondria play active and passive roles in ROS-mediated damage. ROS decrease mitochondrial functionality, decreasing O_2_ usage and ATP generation; and mitochondrial DNA acts as an “*in vivo* dosimeter,” measuring the exposure of a given cell to oxidative damage. Furthermore, mitochondria may also actively produce ROS (34).

Due to the central role of oxidation in the processes mentioned above, anti-oxidant and other absorption mechanisms protect against photoaging and photocarcinogenesis. One such absorptive mechanism is the isomerization of *trans*-urocanic acid into the *cis*- form, which has immunosuppressive properties (35). As stated above, different enzymatic systems (SOD, catalases, peroxidases, GSH and GST) also quench free radicals in different states, reducing UV-mediated oxidation and decreasing their impact on the cell’s DNA (34, 36, 37). The current measurements regulated by the ISO normative to determine the SPF of a given sunscreen do not contemplate the accumulative effect of oxidative damage, particularly in light of the fact that SPF mainly considers the effect of UVB photons, whereas oxidative damage from UVB is much lower than that from UVA radiation.

## UV-Induced Skin Photoaging

The relationship between UV-induced skin erythema and photoaging is well established, and previous reviews in the field cover this aspect in minute detail (38, 39). Repeated erythema causes a wound healing-like behavior, including the onset of scarring-like events that promote ECM remodeling. Some of these events are MMP (matrix metalloproteases) secretion, collagen cross-linking and elastin degradation (17, 40, 41). All these events promote massive tissue remodeling, the onset of wrinkling and the induction of “visible aging.” Photo aging scoring is a key clinical aspect of this process that has been reviewed elsewhere (42–44). The most frequent and best characterized mutation in mitochondrial DNA (mtDNA) and marker of UVA light induced photoaging is a deletion of 4,977 base pairs, called the “common deletion.” UVA radiation generates the common deletion in human fibroblasts through an oxidative mechanism, which depends on the generation of singlet oxygen, other ROS and RNS.

## UV-Induced Immunosuppression

UV-induced immunosuppression is a multi-pronged mechanism that affects different cell types, especially myeloid subtypes, e.g., Langerhans cells (45, 46) and langerin + dendritic cells (9). It also induces the production of immunosuppressive cytokines by keratinocytes (47) and skin macrophages (48). Irradiated myeloid cells undergo abnormal maturation and cannot migrate normally to the lymph nodes, decreasing the skin’s defenses against pathogens (49) which may also lead to imbalanced homeostasis of the skin with resident commensal bacteria (50). Despite the immediate anti-bacterial and anti-viral effect of UV, long-term exposure causes photoimmunosuppression, decreasing the immune system’s ability to promote pathogen clearance, including fungal pathogens, virus and bacteria. There is evidence that UV-mediated LC depletion promotes the recruitment of monocytes and immature dendritic cells that try to compensate the function of LC in the skin (51).

Immunosuppression is a crucial hallmark of cancer (52). Hence, it is possible that a sunscreen with high SPF may not completely prevent photocarcinogenesis. This would be due to the combination of skin damage occurring below MED and immunosuppression, particularly in individuals with genetic susceptibility. It has been well-demonstrated that the ability of sunscreens to prevent immunosuppression is not related to the MED, which is more influenced by UVB than by UVA, whereas UVA is at least as potent as an immunosuppressive agent as UVB [see below and Ref. (53)]; this indicates that MED measurements, which are the basis of SPF determination as per ISO24444:2019, do not correlate with the ability of a sunscreen to prevent immunosuppression.

It is worth noting that approaches that combine light measurements and immunosuppression have been carried out. For example, contact hypersensitivity assays using 2-chloro-l,3,5-trinitrobenzene (TNCB) or 1-fluoro-2,4-dinitrobenzene (DNFB) as irritants were carried out in skin of mice pre-irradiated with different UV wavelengths (54, 55). The efficacy of sunscreens containing UV filters to protect against suppression of CHS induction has been investigated in several studies and revealed that photo-immunoprotection correlated with UVA protection (56, 57). Similar measurements have been performed in humans using nickel as contact allergen on the efferent immune response, which constitute the basis of the Human Immunoprotection Factor, or HIF (58). In humans, two bands that caused immunosuppression were identified, one between 290 and 310 nm (UVB) and one that peaked around 370 nm (UVA). The 370 nm peak, which some authors extend to 380 nm (59), is particularly interesting when discussing oxidative damage.

## Hyperpigmentation and Other Skin Alterations Induced by Visible Light

Pigmentation is induced by UV radiation, but also by visible light (60). In higher skin phototypes, visible light induces more durable photo-pigmentation than UVA irradiation (12). At a mechanistic level, blue light induces melanin production by activating the photoreceptor opsin-3, which acts on the transcription factor Mitf (61), controlling tyrosinase expression and thus melanin production (62). In addition, blue light also generates ROS, which causes photo-oxidative damage to DNA and cellular structures, for example, inducing MMP secretion (27). Photoprotection against visible light cannot be conjoined with UV protection since canonical, FDA-approved UV blockers, e.g., TiO_2_ or ZnO become whitish upon irradiation with visible photons (60), thus becoming unappealing from a cosmetic standpoint. This means that different filters are needed, or the cosmetic formulations of TiO_2_/ZnO need additional inactive ingredients that disguise the whitening of these oxides. Different approaches could be used, from iron oxide (FeO) to natural extracts (see below). Furthermore, sunscreens containing higher concentrations of UV blockers increase the whitish appearance of the skin under visible light, which has unpleasant effects on the customer and a tendency to “sacrifice” better protection for aesthetic reasons.

## Beyond Sun Protection Factor: Biological Actuators vs. Photon Blockers

Accepting the limitations of SPF is a risky proposition due to the exclusion of DNA damage, photoaging and immunosuppressive effects of UV light, as detailed above. However, the current ISO has no wiggle room to incorporate additional parameters. Current organic and inorganic filters do provide considerable protection against such damage, but recent studies have shown that significant additional protection can be achieved by adding other ingredients to sunscreen formulations. These biological filters and/or non-filtering modulating biological molecules can induce additional biological effects, e.g., protection against DNA damage, photoaging or immunosuppression. The major limitation of this approach is that the FDA describes sunscreens as “drugs”,^1^ hence the threshold for the demonstration of safety and efficacy is very high (see discussion of GRASE list in section “Sun Protection Factor and UVA Protection Factor: Are They Still the Benchmark for Photoprotection”). The FDA even acknowledges the limitation of SPF by forcing producing companies to include the following: “Skin Cancer/Skin Aging Alert: Spending time in the sun increases your risk of skin cancer and early skin aging. This product has been shown only to help prevent sunburn, not skin cancer or early skin aging.” In agreement with the previous statement, United States labeling distinguishes clearly between active ingredients (normally ZnO and/or TiO_2_) and inactive ingredients, which usually include oxide solvents, moisturizers, aromatic substances and vitamins.

Conversely, non-US regulations are more flexible because sunscreens are considered cosmetics.^2^ This results in a somewhat wider list of accepted ingredients. However, claims of efficacy need to be backed up by scientific evidence, particularly when it comes to the prevention of immunosuppression.

The figure of “biological filter” emerges from these regulatory differences. A biological filter could be defined as a biological molecule or mixture endowed with or without direct sunscreen ability and able to provide additional beneficial effects. Classical examples include botanical extracts containing anti-oxidant moieties. However, the efficacy of these types of ingredients in preventing photoaging and photoimmunosuppression is usually poorly documented. Topical DNA repair enzymes such as photolyase, endonuclease and glycosylase have also been demonstrated to provide some additional protection, particularly with respect to photoimmunosuppression (63, 64), carcinogenesis (65) as well as polymorphic light eruption (66), though those enzymes provide almost no protection against sunburn.

Over the past 25 years, relevant scientific evidence has grown regarding the efficacy of diverse families of natural compounds, e.g., botanical and non-botanical extracts (Table 1). Non-botanical extracts include fatty acid preparations and probiotics (67). Botanical extracts, e.g., red fruit juices, green tea, coffee, and cocoa preparations, fern leaves extracts, etc., contain vitamin derivatives and large amounts of antioxidant moieties, which reduce the impact of oxidation and inflammation, reducing the onset of photoaging and cancer. Potential active principles include carotenes and lycopenes, xantophylls, vitamins (C, D, and E) and various types of polyphenols. Green tea polyphenols –GTPP– include several species, mainly epigallocatechin –EGC–, epigallocatechin-3-gallate –EGCG–, epicatechin –EC–, and epicatechin-3-gallate –ECG–. They all scavenge reactive oxygen species and boost immunity. ROS scavenging and immune enhancement are related, since oxidation suppresses T cell proliferation and DC function in several contexts (49, 68, 69); hence a decrease in the oxidative threshold enhances immune responses, thereby increasing immune surveillance and reinforcing local anti-tumor responses. In addition, GTPP also decrease oxidation-enhanced protein expression, e.g., metalloproteases, which contribute to skin aging and damage (70). In general, most botanical components endowed with antioxidant capabilities promote skin health (Table 1). Very-well characterized examples include diverse hydrophilic extract of the Mesoamerican fern *Polypodium leucotomos* (PL). PL extracts are effective and safe both topically and orally (71). Assays in mice and humans have demonstrated that one of such extracts (Fernblock^®^) increases MED in spite of having a modest filtering ability (72). The extract also counters the biological effects underlying UV-induced photoaging when administered orally. In mice, it decreases erythema and prevents inflammation, increasing the levels of systemic antioxidant systems, e.g., GSH and GSSG (73). Furthermore, it also decreases inflammation in harsher experimental conditions, e.g., human patients irradiated with UVB light (74). In this regard, oral treatment with PL extracts decreased the deleterious effects of UVB irradiation in human volunteers, including appearance of sunburn cells, DNA damage and inflammatory markers (75). Such treatment also decreased inflammation in human patients undergoing psoralens-UVA therapy (69), a form of treatment for psoriasis, dermatitis, vitiligo, polymorphic light eruption and cutaneous T-cell lymphoma (76, 77). In this context, oral treatment with PL extracts also decreased Langerhans cell depletion (74). Treatment also inhibited the appearance of “common deletion” (CD), which is a mitochondrial DNA deletion that denotes UVA-mediated damage (78). More recent evidence has demonstrated that this type of treatment also protects against the effects of visible (blue) light, notably against pigmentation and skin darkening (79). Treatment also regulates opsin-3 and prevents melanin-dependent photo-oxidation induced by digital screen-generated blue light (80) and prevents visible and infrared-induced skin damage (81).

**TABLE 1:** Photoprotective effects of various natural extracts.

	Extract	Effect	Model	PMID
OXIDATIVE STRESS	PL leaves	Inhibits lipid peroxidation	Hs I.V.	8897589, 12788523
	Pomegranate	Inhibits lipid peroxidation	Mm	20946358
GENOMIC DAMAGE	Green Tea (GTPPs)	Decrease CPD	Mm	19020550
		Increase NER gene expression	Mm	20103727
	PL leaves	Reduces levels of 8-oxo-G	Mm	19808641
		Reduces DNA mutation burden	Mm	19808641
		Inhibits CPD formation	Mm	19808641
			Hs	15583582
		Reduces mitochondrial CD	Hs	20159320
	Pomegranate	Reduces levels of 8-oxo-G	Mm	20946358
		Inhibits CPD formation		
UV-ECM DAMAGE	GTPPs	Reduce MMP-2,-9 expression	Mm	16317135
		Enhance TIMP expression		
	PL leaves	Increases collagen expression (I, III, V)	I.V.	19373483
		Inhibits MMP-1	I.V.	19373483
		Increases TIMP	I.V.	19373483
INFLAMMATION	Green Tea Polyphenols	Induce IL-12 secretion	I.V.	18179621
		Inhibit AP-1 and NF-κB	Mm	14681684
	PL leaves	Inhibits TNF-α, iNOS, AP-1, NF-κB expression	I.V.	17845214
		Increases IL-10 expression	I.V.	10928072
		Inhibits leukocyte extravasation	Mm	18312382
		Inhibits COX-2, PGE2 expression	Mm	19808641
			Hs	28341348
IMMUNO-SUPPRESSION	PL leaves	Inhibits *trans*-UCA isomerization	I.V.	16388959
		Inhibits glutathione oxidation	Mm	18312382, 22776002
		Prevents eLC depletion	Mm	14699363
			Hs	

*PL, Polypodium leucotomos; CPD, Cyclobutane pyridimide dimer; NER, Nuclear excision repair; CD, common deletion; 8-oxo-G; MMP, matrix metalloprotease; TIMP, tissue inhibitor of metalloprotease; TNF, tumor necrosis factor; iNOS, inducible nitric oxide synthase; COX, Cyclooxygenase; PGE, prostaglandin E; UCA, urocanic acid; eLC, epidermal Langerhans cells; 8-oxo-G, 8-hydroxy-2′-deoxyguanosine; Hs, Homo sapiens; I.V., in vitro; Mm, Mus musculus.*

Evidence of the efficacy of fern extracts has been obtained at a molecular level, as their antioxidant moiety also inhibited *trans*-urocanic isomerization (82). At a cellular level, treatment with PL extracts blocked the activation of oxidative pathways, blocking DNA damage and promoting its repair (83), and impaired MMPs secretion and matrix remodeling (84). Finally, it efficiently prevented Langerhans cells depletion in humans and rodents (69, 73, 85), leading to decreased inflammation when administered orally (reviewed in Ref. 86).

Recent evidence indicates that the addition of PL extracts to topical formulations with traditional inorganic/organic filters can increase the formulations’ MED, DNA protection, immunoprotection (LC and HIF), and ability to reduce photoaging and pigmentation response (72, 87).

It is important to note that most of these compounds have limited effect on the SPF *per se*; hence they would be considered “inactive ingredients” from a FDA perspective. However, the increasing amount of scientific evidence supporting the efficacy of this type of approach suggests a potential benefit from their inclusion in sunscreens. The peer-reviewed studies regarding the efficacy of natural compounds as photoprotectors have reached a scientific standard that would support the planning and execution of another meeting, in which a large community of experts in the field, including molecular biologists, immunologists, and clinical practitioners (mainly dermatologists and oncologists) review the possible advantages of including natural extracts in topical sunscreen formulations. A new consensus parameter for evaluating photoprotection efficacy could emerge from such a meeting to complement SPF, providing additional information in an easy to understand manner that would allow consumers to make more informed decisions regarding photoprotection. This is particularly important if we consider that photoprotective measures have two tiers of potential users: the mainly healthy individual who purchases sunscreen for outdoor activities; and the patients under specialized care due to pre-existing skin conditions, e.g., vitiligo, hyperpigmentation, photodermatoses, psoriasis, actinic keratoses and skin cancer, rosacea, atopic dermatitis, advanced skin aging, etc. Sunscreens are necessary for every individual, but formulations with effective activity against a wide range of damage are particularly important for patients suffering from chronic skin diseases and sun-related conditions, which comprise a significant percentage of society. Some examples of prevalence are 0.1–2% for vitiligo (88); 0.2–5% for psoriasis (89); and 1–2% for non-melanoma skin cancer.^3^ Most of these patients are under dermatologist care. Thus, the decision to recommend one sunscreen or another can be guided by the specialist, who has a better understanding of the different factors and/or beneficial effects of inactive ingredients. In order to achieve this, it is essential that care providers have specific training (“photo-education”) that enables them to interpret the pertinent information regarding the additional beneficial effects of the composition of sunscreens in order to make informed recommendations. In this context, providing additional information regarding the biological effects of inactive ingredients can ultimately contribute to the wellbeing of patients.

## Future Challenges

In addition to the evident need to generate a photoprotection index that is more inclusive than SPF, the field faces additional challenges. One is the growing evidence that UV- and visible light-mediated skin oxidation alters the balance of skin homeostasis with symbiotic microbiome. UV and ROS generation induce alterations in the composition of the skin microbiome, destabilizing skin immune homeostasis and increasing the prevalence of other skin infections, e.g., herpesvirus (90). Such an imbalance may also accelerate photocarcinogenesis (91). For example, *Cutibacterium acnes* produce less porphyrins when irradiated with UVB photons (92), which may lead to decreased inflammatory responses (93). Other symbiotic yeast and bacteria are also affected by UV radiation, unbalancing the skin microbiome and favoring the onset of skin cancer, particularly those related to virus, e.g., HPV and Merkel cell polyomavirus (94). Intriguingly, recent evidence indicates that a commensal strain of *Staphylococcus epidermidis* protects against skin carcinogenesis in a mouse model (95) and a normal skin microbiota reduces photoimmunosuppression (50).

Another important issue is that demographic studies indicate that more and more population is concentrating in large cities, which increases skin exposure contaminants, e.g., combustion derivatives. The WHO has estimated that over 4 million people die yearly as a result of air contamination featuring diverse types of pollutants, e.g., particulate matter, nitrogen dioxide, sulfur dioxide, black carbon, carbon monoxide, and ground-level ozone (96). These pollutants act at three levels: they generate free radicals and oxidative damage; they cause, or amplify, inflammation; and they compromise the integrity of the skin barrier function. Other epithelia are also compromised, e.g., the lungs. Particulate matter is particularly harmful in this respect, including ultrafine particles smaller than 100 nm. These can penetrate skin barriers and have systemic effects, including oxidation and inflammation. Likewise, polycyclic aromatic hydrocarbons, dioxins and ozone can produce oxidative damage and inflammation, amplifying the effects of skin unprotected against to UV radiation. Exposure to these pollutants can have additive or exponential effects that would increase UV-induced photo-damage, either during exposure (UV may ionize some of these compounds, increasing their toxicity) or post-exposure (pollutants on UV-induced damaged skin may contribute to infection, slow healing or local immunosuppression). Again, SPF does not account for these amplifying/accumulative effects, which nonetheless will inevitably affect sunscreen end users. These novel effects will also need to be taken into account when developing the next generation of photoprotective sunscreens and the measurements that will define their activity.

Additional challenges remain, including patient education and the incorporation of nanotechnologies to skin care, which constitute exciting new frontiers in the field.

## Conclusion

Despite increased awareness and use of photoprotection sunscreens, cases of skin cancer continue to grow worldwide. Photoprotection efficacy of sunscreens is measured and communicated primarily by SPF, and to a lesser extent UVA-PF and critical wavelength. These measurements do not, however, take into consideration damage caused to the skin beyond MED-related measurements such as DNA damage, photoaging and immunosuppression. In addition, there is a large and growing body of evidence indicating that sub-MED doses of UV radiation can indeed accelerate photoaging and mediate immunosuppression and DNA damage. Current SPF and UVA-PF ratings do not contemplate damage from other wavelengths such as visible and IR ranges.

Given the limitations of current measurements, there is an emergent need for complementary methods to assist specialists in determining the most suitable sunscreen for their patients, especially those suffering from chronic skin diseases and sun related conditions. In addition a new comprehensive final index including ISO-compliant SPF measurements but also determination of other levels of protection against solar radiation could facilitate better overall understanding of sunscreen efficacy amongst the general public.

Current organic and inorganic filters provide considerable protection against sun damage, but recent studies have shown that significant additional protection can be achieved by adding other ingredients to sunscreen formulations. These additional components do not need to be physical filters, but they must induce additional biological effects, e.g., protection against DNA damage, photoaging or immunosuppression. Additional components take the form of botanical or non-botanical extracts endowed with antioxidant, DNA protective and immunostimulating properties. A plethora of scientific evidence supports the potential benefit of inclusion of these types of extracts in commercial sunscreens and oral supplements with no additional risk to the overall health of the end users.

## Data Availability Statement

The original contributions presented in the study are included in the article/supplementary material, further inquiries can be directed to the corresponding author.

## Author Contributions

SG conceptualized and prepared the agenda that was discussed during the meeting. All rest of the authors contributed equally to this article and their names are listed in alphabetical order.

## Conflict of Interest

SG is a consultant for Cantabrialabs, which produces Fernblock^®^. C-LG and FX are members of Asian Medical Advisory Board for Cantabrialabs. BB is a consultant for Ferndale. HL has served as an investigator for Incyte, La Roche-Posay, Pfizer, PCORI, as a consultant for Pierre Fabre, ISDIN, Ferndale, La Roche-Posay, Beiersdorf, and as a non-product specific speaker for La Roche-Posay and Cantabria Labs. FX is a consultant for Skinceuticals, La Roche-Posay, Avene, Bioderma, CeraVe, Cantabria Labs, and Winona. The remaining authors declare that the research was conducted in the absence of any commercial or financial relationships that could be construed as a potential conflict of interest.

## Publisher’s Note

All claims expressed in this article are solely those of the authors and do not necessarily represent those of their affiliated organizations, or those of the publisher, the editors and the reviewers. Any product that may be evaluated in this article, or claim that may be made by its manufacturer, is not guaranteed or endorsed by the publisher.

## Footnotes

1
https://www.fda.gov/regulatory-information/search-fda-guidance-documents/labeling-and-effectiveness-testing-sunscreen-drug-products-over-counter-human-use-small-entity#_Toc281970892

2
https://eur-lex.europa.eu/legal-content/EN/TXT/?uri=CELEX:02009R1223-20190813

3
https://www.cancer.net/cancer-types/skin-cancer-non-melanoma/statistics
